# BSMV-mediated genome editing exhibits host-specific heritability: germline transmission in barley and somatic edits in *Nicotiana benthamiana*

**DOI:** 10.1186/s12870-026-08866-3

**Published:** 2026-05-12

**Authors:** Pankaj K. Bhowmik, John T. Williams, Brittany Polley, Naichong Chen, Naga Rajitha Kavuri, Wen Zang, Abdellah Barakate, Hui Yang, Murali Krishna Narra, Aaron D. Beattie, Colby Starker, Daniel F. Voytas, Can Baysal

**Affiliations:** 1 National Research Council of Canada, Saskatoon, SK S7N 0W9 Canada; 2 Department of Horticultural Sciences, University of Florida, Gainesville, FL 32611 USA; 3 Crop Transformation Center, University of Florida, Gainesville, FL 32611 USA; 4 Department of Plant Sciences, University of Saskatchewan, Saskatoon, SK S7N 5A8 Canada; 5 Cell and Molecular Sciences, The James Hutton Institute, Invergowrie, Dundee, Scotland, UK; 6 Center for Precision Plant Genomics, Department of Genetics, Cell Biology and Development, University of Minnesota, St. Paul, MN 55108 USA

**Keywords:** Barley, CRISPR/Cas, *N. benthamiana*, Plant RNA viruses, Viral Promoter, Virus-induced heritable genome editing

## Abstract

**Background:**

Plant RNA virus–mediated guide RNA (gRNA) delivery represents a transformative advance in genome editing technologies. Unlike conventional transformation methods that rely on labor-intensive tissue culture and regeneration for each individual gRNA delivery, viral vectors can rapidly and systemically transmit gRNAs into pre-established Cas-expressing plants, providing an accelerated route for functional genomics and trait discovery directly *in planta*. However, key design parameters, including subgenomic promoter choice, transcript architecture, and their effects on viral fitness and editing outcomes, remain to be elucidated for most viral platforms.

**Results:**

We developed five Barley stripe mosaic virus (BSMV) vectors, each with distinct subgenomic promoter elements to drive single gRNA expression. These were initially evaluated in Cas9-expressing transgenic *Nicotiana benthamiana* plants targeting the *Phytoene desaturase* (*PDS*) gene to compare their editing efficiencies. Single gRNAs expressed under the duplicated γb subgenomic promoter or when fused directly to the γb genome achieved the highest mutation frequencies (up to 90% at 60 days post-inoculation), whereas β1- and β2-driven sgRNAs produced delayed and reduced editing. Thus, promoter selection critically determines gRNA accumulation and the efficacy of BSMV-mediated genome editing. The top-performing design was then applied to Cas9-expressing barley (*Hordeum vulgare*) targeting *HvCMF7* (conferring green-white variegation) and *HvGW2.1* (impacts grain width and weight). BSMV spread systemically throughout barley, inducing somatic and heritable mutations at frequencies up to 100%, with virus-free edited progeny. In contrast, despite robust somatic editing in *N. benthamiana*, no heritable mutations were detected indicating species-dependent limitations in germline transmission.

**Conclusion:**

Our systematic comparison of subgenomic promoter architectures establishes clear design principles for optimizing viral vector–mediated delivery. Promoter choice and transcript structure critically shape editing efficiency and viral stability. The host-specific boundary for germline editing, defined by efficient heritable editing in barley but not *N. benthamiana*, highlights where BSMV offers advantages and where alternative vectors or hybrid strategies are required, guiding rational platform selection for diverse crop species and applications. Collectively, these findings establish BSMV as a promising next-generation vector for rapid, tissue culture–free, and transformation-independent genome editing in cereals and other recalcitrant monocots.

**Supplementary Information:**

The online version contains supplementary material available at 10.1186/s12870-026-08866-3.

## Background

Recent advances in plant molecular biology have established plant RNA viruses as powerful, tissue culture–independent delivery systems. Their capacity for autonomous replication, high levels of transient expression, and systemic movement through host tissues enables rapid functional genomics directly *in planta*, bypassing the need for stable transformation or lengthy regeneration processes [1, 2]. A diverse suite of viral vectors has since been engineered to enable gene silencing, genome editing, and transient protein expression. Most RNA viruses accommodate compact cargos (typically <2 kb) such as single guide RNAs (sgRNAs) and small proteins. These include Bamboo mosaic virus (BaMV), Barley stripe mosaic virus (BSMV), Barley yellow striate mosaic virus (BYSMV), Brome mosaic virus (BMV), Cucumber mosaic virus (CMV), Foxtail mosaic virus (FoMV), Tobacco rattle virus (TRV), Tobacco ringspot virus (TRSV), Potato virus X (PVX) and Sugarcane mosaic virus (SCMV) [2–16]. Larger-genome RNA viruses such as Citrus tristeza virus (CTV) provide distinct tissue tropisms and extended expression windows that can be useful for certain phloem-targeted applications, while plant DNA replicons (for example, geminiviral systems) offer alternative routes to amplify donor templates or compact nucleases [17–19].

Dicot systems have recently achieved tissue culture–free and transgene-free heritable editing by co-delivering compact nucleases and their sgRNAs directly from RNA viral genomes [20, 21]. These studies demonstrate that viral RNAs can indeed reach germline cells when cargo design, expression balance, and host compatibility are carefully optimized. Notably, engineered TRV and TRSV platforms have successfully delivered miniature genome editors, such as TnpB and compact Cas12-family nucleases, to achieve germline edits in wild-type Arabidopsis, *N. benthamiana*, and tomato [20, 21].

Among monocot-adapted viral vectors, BSMV, a positive-sense, tripartite RNA virus (α, β, γ), has emerged as a leading platform for genome editing in cereals because of its robust replication and systemic spread in grasses (particularly in barley, wheat, and oat) which enable efficient sgRNA delivery to Cas-expressing hosts [8–10]. BSMV contains three genomic RNAs: RNAα and RNAγ encode the replicase subunits αa and γa that are translated directly from their genomic RNAs and together are required for viral RNA replication, whereas RNAβ encodes the triple gene block (TGB) movement proteins and coat protein that are essential for cell‑to‑cell and systemic movement in plants [22–24]. Subgenomic mRNAs arise from native promoters on RNAβ and RNAγ to express TGB1 (RNAβ1), TGB2/3 (RNAβ2), and γb (RNAγ), and promoter identity with flanking context determinants governs transcript abundance and timing across these loci. Yb protein promotes viral cell-to-cell movement, TGB modules mediate movement for multiple plant virus genera and require precise stoichiometry among TGB1/2/3, which makes expression timing and levels central to preserving efficient cell-to-cell and systemic spread during vectorized cargo delivery [23–25]. Consistent with these mappings, γb and β1 generally accumulate at higher levels than β2, with γb expression supported by a compact promoter showing more constitutive behavior, while β1/β2 are more transient and temporally coordinated to balance movement‑protein stoichiometry [26–28].

Despite this mechanistic understanding, key design parameters governing BSMV performance remain to be defined particularly how subgenomic promoter (SGP) element’s identity, positioning, and transcript architecture influence sgRNA abundance, viral fitness, systemic spread, editing frequency, and host-specific heritability. To address these gaps, we engineered five sgRNA-expression architectures for systematic comparison: four SGP‑driven designs (β1 SGP, β2 SGP, truncated β2 SGP and γb SGP) and a fifth design in which the sgRNA was fused directly to the γb genomic RNA context to test a noncanonical transcript architecture, an approach consistent with prior BSMV vector engineering strategies. All five architectures were first tested in Cas9-expressing *N. benthamiana* to determine viral stability, systemic movement, quantify somatic editing and screen for heritable transmission. The top-performing configuration was then translated to Cas9-expressing barley to edit two loci with clear phenotypic or agronomic relevance: (i) *HvCMF7* (*Albostrians*), a chloroplast biogenesis gene yielding a scorable green–white variegation that sensitively reports somatic and heritable events; and (ii) *HvGW2.1* (*Grain Width and Weight 2.1*), which impacts grain yield, size and weight.

Our findings demonstrate that BSMV vector performance is strongly dependent on promoter architecture. Single gRNAs expressed via direct fusion to the γb genome or the duplicated γb subgenomic promoter yielded the highest somatic editing frequencies in both *N. benthamiana* and barley. In contrast, vectors using the truncated β1 and β2 subgenomic promoters supported functional but slower editing kinetics. The full-length β2 promoter was unable to express the *PDS* sgRNA and failed to infect *N. benthamiana*. Importantly, although systemic RNA delivery and high-frequency somatic mutagenesis were achieved in both species, heritable edits were exclusively recovered in barley. This indicates a host-specific barrier, likely due to antiviral defense mechanisms restricting BSMV access to reproductive tissues in dicots. Together, these results establish clear design principles for BSMV-based genome editing, reveal host-specific limits on germline transmission, and provide a foundation for next-generation viral systems aimed at eliminating tissue culture requirements in cereal crop biotechnology.

## Materials and methods

### Plant materials

Standard *Agrobacterium*-mediated transformation of *Hordeum vulgare* was conducted using immature embryos 12–14 days post-pollination as explants according to the method described by [29]. Briefly, the pBract214m-HvCas9-OsU6pgRNA construct was introduced into *Agrobacterium* strain AGL1 and used to integrate HvCas9 (*Hordeum vulgare*) codon optimized *Streptococcus pyogenes* Cas9) into the wild-type Canadian barley line TR09398 genome. All T2 plants used in this study were derived from a single Cas9-expressing T1 line, and the presence of at least one Cas9 copy was confirmed in all analyzed plants. Wild-type *N. benthamiana* plants (for viral inoculum preparation) and SpCas9-expressing *N. benthamiana* plants (for virus-mediated genome editing) were grown in a controlled light room at 24 °C with a 16/8 h light/dark photoperiod and a light intensity of 80–100 µmol m⁻² s⁻¹. Cas9-expressing barley plants were cultivated in a growth chamber (Conviron) at 22/18°C (day/night), 50% relative humidity, and a 12/12 h light/dark cycle with 350 µmol m⁻² s⁻¹ light intensity. Offspring from virus-infected plants were maintained under the same controlled conditions.

### Construction of BSMV derived viral vectors

Five Barley stripe mosaic virus gamma (BSMVγ) vectors were generated, each incorporating distinct subgenomic promoters to express *NbPDS* gRNA. Using Golden Gate cloning, SapI restriction sites were inserted flanking the viral promoters and sgRNA insertion regions in the viral genome. Forward and complementary reverse oligonucleotides along with overhangs were annealed and inserted into BSMVγ vector (pYL157) flanking 3′ region of the sgRNA scaffold. The annealed fragments were assembled into the BSMV cloning vector pYL157 via Golden Gate Assembly. The reaction contained 0.5 µl of each PCR product, 1 µl of pYL157 (50 ng), 0.5 µl SapI, 0.5 µl T4 DNA ligase, 2 µl T4 ligase buffer, and 14.1 µl H₂O. The cycling protocol was 37 °C for 5 min and 16 °C for 10 min (30 cycles), followed by 37 °C for 10 min and 80 °C for 5 min. Single gRNAs targeting *HvCMF7* and *HvGW2.1* were selected based on prior successful genome editing studies in barley [7, 10, 30]. For BSMV-γ-sgRNA expression constructs, complementary oligonucleotides were annealed and cloned into the BSMV-γ vector (pGY036) via Golden Gate Assembly using AarI. BSMV constructs used in this study are based on the ND18 strain, and all vector modifications, including γb promoter architectures, were generated using this ND18 backbone. All constructs were sequence-verified via Sanger sequencing using insert-specific primers. Primers used in cloning are listed in Table S1, and BSMV vectors have been deposited in Addgene.

### Agroinfiltration of viral T-DNAs to *N. benthamiana* leaves, viral sap preparation and mechanical inoculation

The BSMVα (pYL155), BSMVβ (pYL156), and five distinct BSMVγ-sgRNA plasmids (pCB008–pCB012) were transformed individually into *A. tumefaciens* GV3101 using the freeze–thaw method [31]. Agrobacterium cultures were grown in LB medium supplemented with gentamicin (50 mg L⁻¹) and kanamycin (50 mg L⁻¹) at 28 °C overnight. Cells were pelleted (3000 × g, 10 min) and resuspended in infiltration buffer (10 mM MgCl₂, 10 mM MES [pH 5.8], 0.15 mM acetosyringone). Each engineered BSMVγ vector was combined with the α and β components, and the mixtures (final OD₆₀₀ = 0.8) were agroinfiltrated into 5–6-week-old *N. benthamiana* leaves. For the *N. benthamiana* experiments, each BSMV construct was evaluated in individual plants. Three leaves per plant were agroinfiltrated, and at 30 and 60 dpi, three upper systemic leaves per plant were sampled for mutation analysis. For the full-length β2 construct, three independent plants were tested. For progeny analysis, seeds were collected from infected parental plants, with approximately 200 seeds germinated per plant. For BSMV-mediated genome editing in barley, the α, β, and γ-sgRNA plasmids targeting *HvCMF7* (pCB032) and *HvGW2.1* (pCB131) were transformed separately into *A. tumefaciens* GV3101 as described above. Infiltrated *N. benthamiana* leaves were harvested at 10 dpi, ground on ice in 20 mM Na-phosphate buffer (pH 7.2) containing 1% celite or silicon carbide powder (4 ml buffer per 2 g fresh tissue), and viral sap was stored at − 80 °C for further use. For barley inoculation, 200 µl of viral sap was applied to the two to three-leaf stage (two-week-old seedlings) by gently rubbing with gloved fingers until a slight squeak signaled effective abrasion Fig. S1.

### Analysis of editing efficiencies

Genomic DNA was extracted from systemically infected upper 3 leaves and non-infected control plants using the DNeasy Plant Pro Kit (QIAGEN, Cat. No: 69206) or the DNeasy Plant Mini Kit (QIAGEN, Cat. No. 69106), following the manufacturer’s protocols. sgRNA target sites were amplified by PCR using gene-specific primers (Table S1). PCR products were digested with NcoI restriction enzymes that recognize wild-type sequences but are disrupted by mutations to generate polymorphic band patterns indicative of editing.

The digested products were resolved on agarose gels, and band intensities were quantified to estimate editing frequencies. Sanger sequencing of PCR products was performed (Eurofins Genomics LLC) to confirm edits and to quantify indel frequencies using the ICE CRISPR Analysis Tool [32]. For heritability analysis, progeny from highly edited lines were grown in soil, and genomic DNA from individual seedlings was similarly analyzed by CAPS and sequencing.

## Results

### Design of BSMV γ-vectors carrying distinct sub-genomic promoter elements for sgRNA expression

**Fig. 1:**
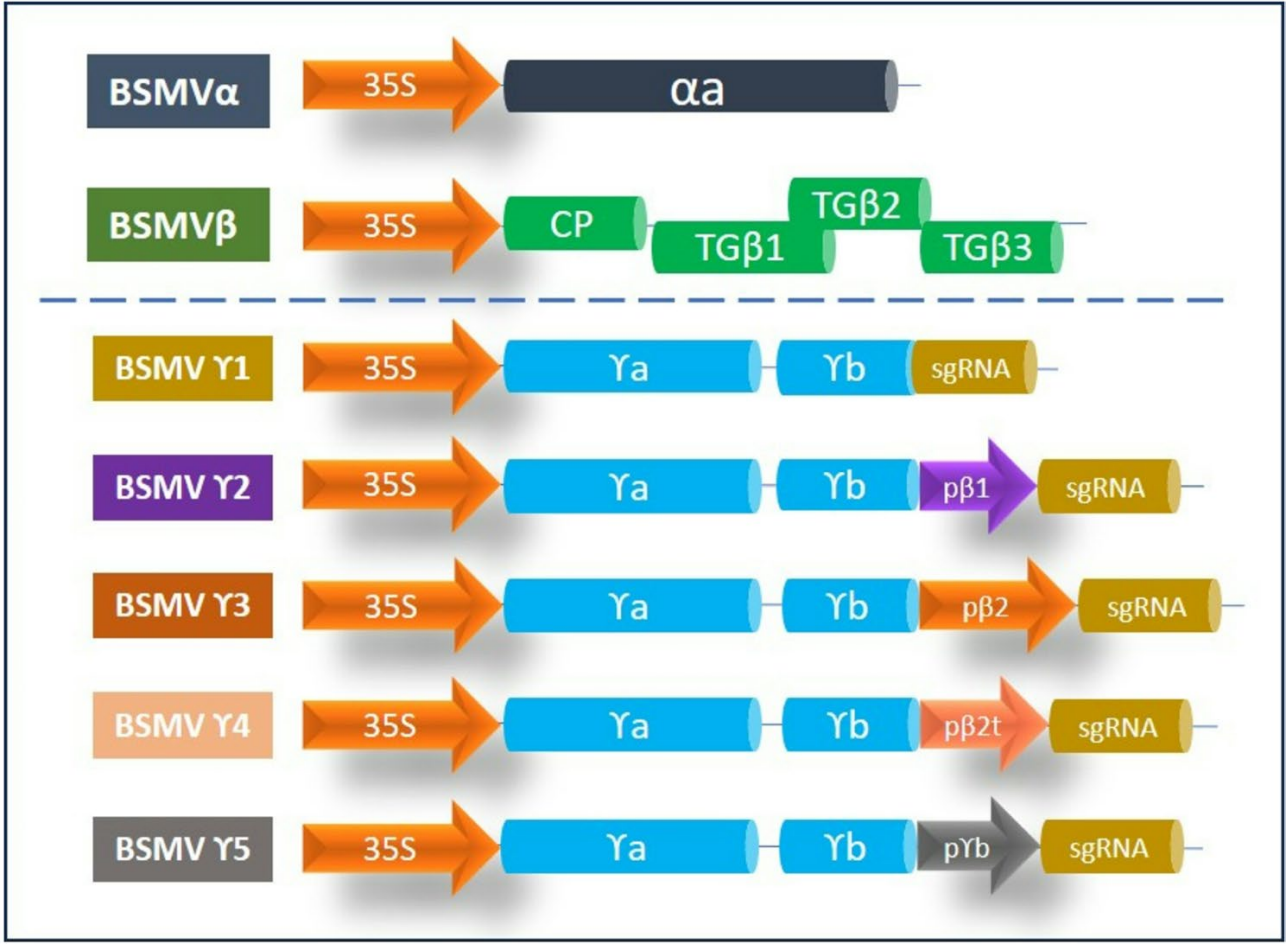
BSMV vector architecture and subgenomic promoter elements tuning for sgRNA delivery. Schematic of BSMV T-DNA constructs driven by the CaMV 35S promoter: RNAα encodes the helicase-like replicase subunit αa; RNAβ encodes coat protein (CP) and the triple gene block movement proteins (TGB1/2/3); and RNAγ encodes the polymerase-like replicase subunit γa and the accessory protein γb, with γb translated from sgRNAγ. The β-derived movement proteins are normally expressed from distinct native subgenomic mRNAs, with TGB1 from sgRNAβ1 and TGB2/3 from sgRNAβ2, providing the baseline map used to position alternative native promoters for transcribing vectorized cargos. The five BSMV γ-vectors were designed to deliver a sgRNA: (1) direct fusion to the γb genome (γb-sgRNA), (2) β1 SGP-driven expression (pβ1-sgRNA), (3) full length β2 SGP-driven expression (pβ2-sgRNA), (4) truncated β2 SGP-driven expression (pβ2t-sgRNA), and (5) γb SGP-driven expression (pγb-sgRNA)

**Fig. 2:**
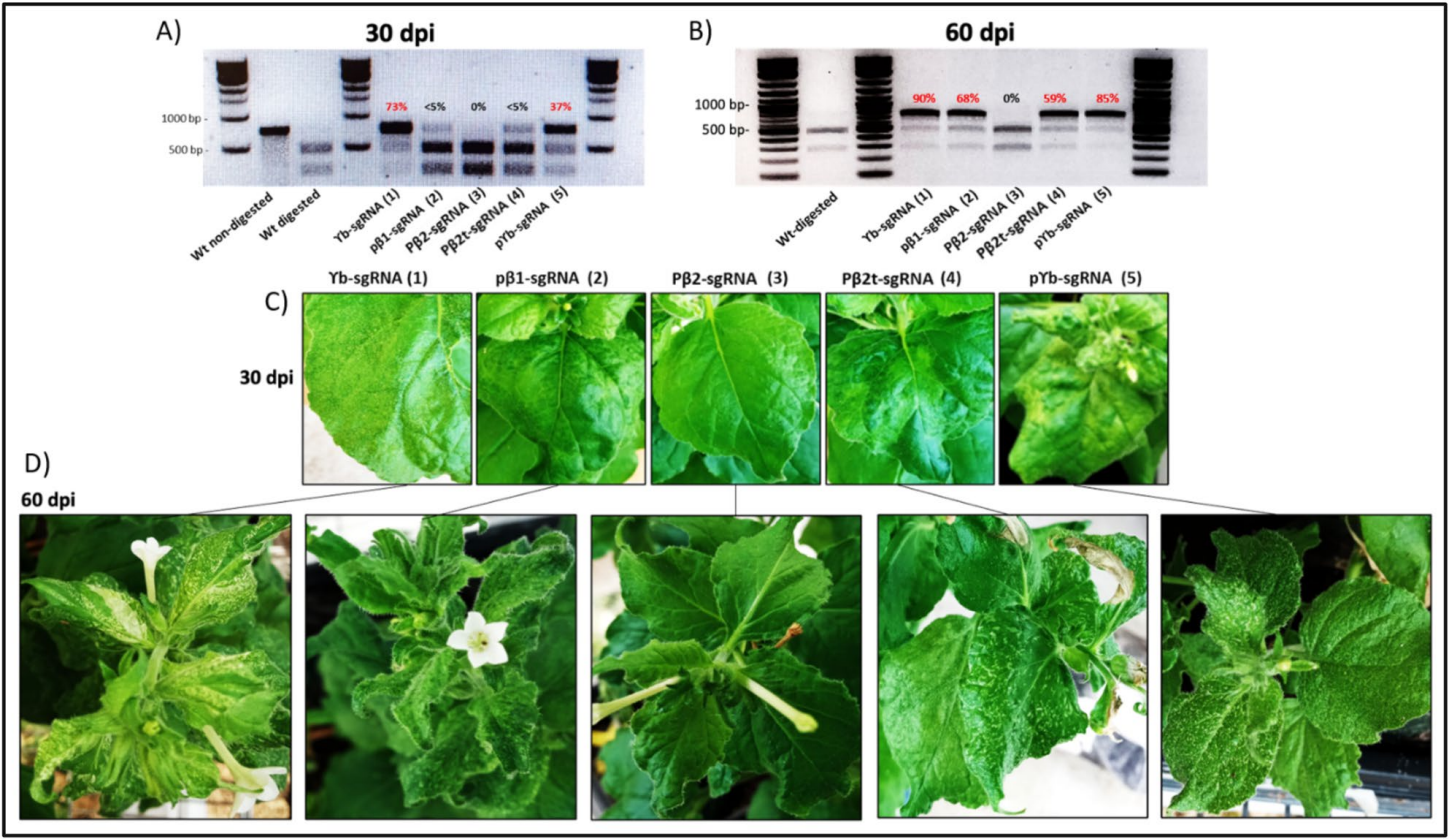
Editing outcomes and phenotypes from BSMV vectors expressing sgRNA under distinct BSMV subgenomic promoters.​​ **A** CAPS assay at 30 days post-inoculation (dpi) showing wild-type non-digested and digested controls alongside five promoter variants: γb-sgRNA (1), pβ1-sgRNA (2), pβ2-sgRNA (3), pβ2t-sgRNA (4), and pγb-sgRNA (5), with red percentages indicating estimated indel frequencies derived from Sanger sequencing and restriction digest–based readout (CAPS) of the target region. Observed estimates at 30 dpi are approximately 73% (γb), < 5% (pβ1), 0% (pβ2), < 5% (pβ2t), and 37% (pγb), as annotated above each lane.​​ **B** CAPS and Sanger sequencing assay at 60 dpi for the same samples showing increased editing for several promoter configurations, with annotated estimates of approximately 90% (γb), 68% (pβ1), 0% (pβ2), 59% (pβ2t), and 85% (pγb). Lane order and controls mirror panel A to facilitate direct comparison across time points.​​ **C** Representative leaves at 30 dpi illustrating foliar phenotypes associated with each promoter variant (1–5 in the order defined above), including varying degrees of mosaic symptoms consistent with systemic vector activity and editing frequencies. **D** Representative plants at 60 dpi displaying intensified foliar phenotypes for several promoter variants, with expanded mosaic symptoms and PDS bleaching phenotypes in systemic tissues relative to 30 dpi. Images are arranged left-to-right in the same sample order (1–5) to align phenotypic progression with the corresponding editing readouts in panels A and B. Each BSMV construct was evaluated in individual plants. Three leaves per plant were agroinfiltrated, and at 30 and 60 dpi, three upper systemic leaves per plant were sampled for mutation and phenotypic analyses. Detailed sequencing analysis of BSMV-induced mutations in systemic leaves of *N. benthamiana* at 30 and 60 dpi is shown in Figs. S2 and S3

To systematically evaluate how subgenomic promoter architecture influences sgRNA-mediated editing, five BSMV γ-vectors were engineered to deliver a sgRNA targeting the *NbPDS* gene (sgRNA sequence: TTTGGTAGTAGCGACTCCATG) in Cas9-expressing *N.* *benthamiana* plants. The five architectures comprised: (1) direct fusion to the γb genome (γb-sgRNA), (2) β1 SGP-driven expression (pβ1-sgRNA), (3) full length β2 SGP-driven expression (pβ2-sgRNA), (4) truncated β2 SGP-driven expression (pβ2t-sgRNA), and (5) γb SGP-driven expression (pγb-sgRNA) (Fig. 1**)**. Plants were agroinfiltrated with recombinant BSMV constructs (BSMV γ-vectors along with BSMVα, BSMVβ), and upper systemic leaves were sampled at 30- and 60-days post-infiltration (dpi) to evaluate viral movement, sgRNA delivery, and genome editing efficiency. Viral movement and systemic infection were assessed based on phenotypic observations and editing outcomes. Four of five BSMV constructs established systemic infection in *N. benthamiana*, as confirmed by the somatic mutations 30- and 60- dpi (Fig. 2A, B), except for the PDS sgRNA expressed under the full-length β1 promoter (construct 3). Photobleaching indicative of PDS disruption was first visible at 30 dpi in plants inoculated with γb-sgRNA (construct 1) and pγb-sgRNA (construct 5), showing pale-yellow phenotype on newly emerged leaves (Fig. 2C). In contrast, plants inoculated with β1- and β2-driven constructs (constructs 2 and 4) displayed minimal or no photobleaching at this early time point, consistent with low editing efficiencies observed by CAPS analysis (Fig. 2A). By 60 dpi, photobleaching intensified and expanded in plants inoculated with γb- and pγb-based constructs, with large sectors of tissue showing complete loss of green pigmentation (Fig. 2D). Plants inoculated with pβ1-sgRNA and truncated pβ2-sgRNA exhibited moderate photobleaching by 60 dpi, consistent with increased but comparatively lower editing efficiencies (Fig. 2B). Detailed sequencing analysis of BSMV-induced mutations in systemic leaves of *N. benthamiana* at 30 and 60 dpi is provided in Supplementary Figures S2 and S3. Wild-type (non-inoculated) and mock-inoculated Cas9 plants remained fully green throughout the experiment, confirming that photobleaching was specifically associated with BSMV-delivered *PDS* gRNA and Cas9 activity.

### BSMV-mediated somatic editing in *N. benthamiana* is promoter-dependent and time-variable

To quantify editing efficiency, genomic DNA was extracted from upper systemic leaves at 30 and 60 dpi, and the *NbPDS* target region was PCR-amplified, subjected to NcoI restriction digestion (CAPS assay) and Sanger sequenced. The sgRNA target site contains an NcoI recognition sequence (CCATGG), and successful editing events disrupt this site, yielding undigested PCR products that can be visualized by gel electrophoresis. At 30 dpi, γb-sgRNA (construct 1) and pγb-sgRNA (construct 5) produced the highest editing frequencies, with 73% and 37% of PCR products remaining undigested, indicating robust early editing **(**Fig. 2A, C**)**. In contrast, pβ1-sgRNA (construct 2) and pβ2t-sgRNA (construct 4) showed minimal editing (< 5% undigested), demonstrating delayed sgRNA accumulation or activity at this timepoint **(**Fig. 2A, C**)**.

By 60 dpi, editing efficiency increased across all constructs, consistent with progressive viral replication and sgRNA accumulation in systemic tissues. Yb-sgRNA (construct 1) maintained the highest editing frequency at approximately 90% (Fig. 2B, D), indicating sustained and efficient sgRNA delivery and Cas9 activity. pβ1-sgRNA (construct 2) reached 68% editing (Fig. 2B, D), demonstrating substantial improvement over the 30 dpi timepoint and confirming that β1-driven sgRNA expression can support robust editing with delayed kinetics. pβ2-sgRNA (construct 3) exhibited no detectable editing (0%) (Fig. 2B, D), suggesting that the duplicating β2 promoter context may impair sgRNA stability, processing, or accumulation. Truncated pβ2t-sgRNA (construct 4) achieved 53% editing (Fig. 2B, D), indicating that the shorter β2 promoter context is functional but yields lower sgRNA output than γb-fusion or β1-driven designs. pγb-sgRNA (construct 5) produced 85% editing (Fig. 2B, D), demonstrating that γb SGP-driven expression is highly efficient and nearly equivalent to direct γb-fusion in terms of final editing frequency, though with slightly delayed onset relative to construct 1. Given the strong editing performance achieved with γb-based constructs in *N. benthamiana*, we next applied the most efficient design to Cas9-expressing barley to evaluate its potential to induce somatic and heritable genome edits in a monocot host.

### BSMV-mediated sgRNA delivery induces somatic and heritable mutations in transgenic barley

**Fig. 3:**
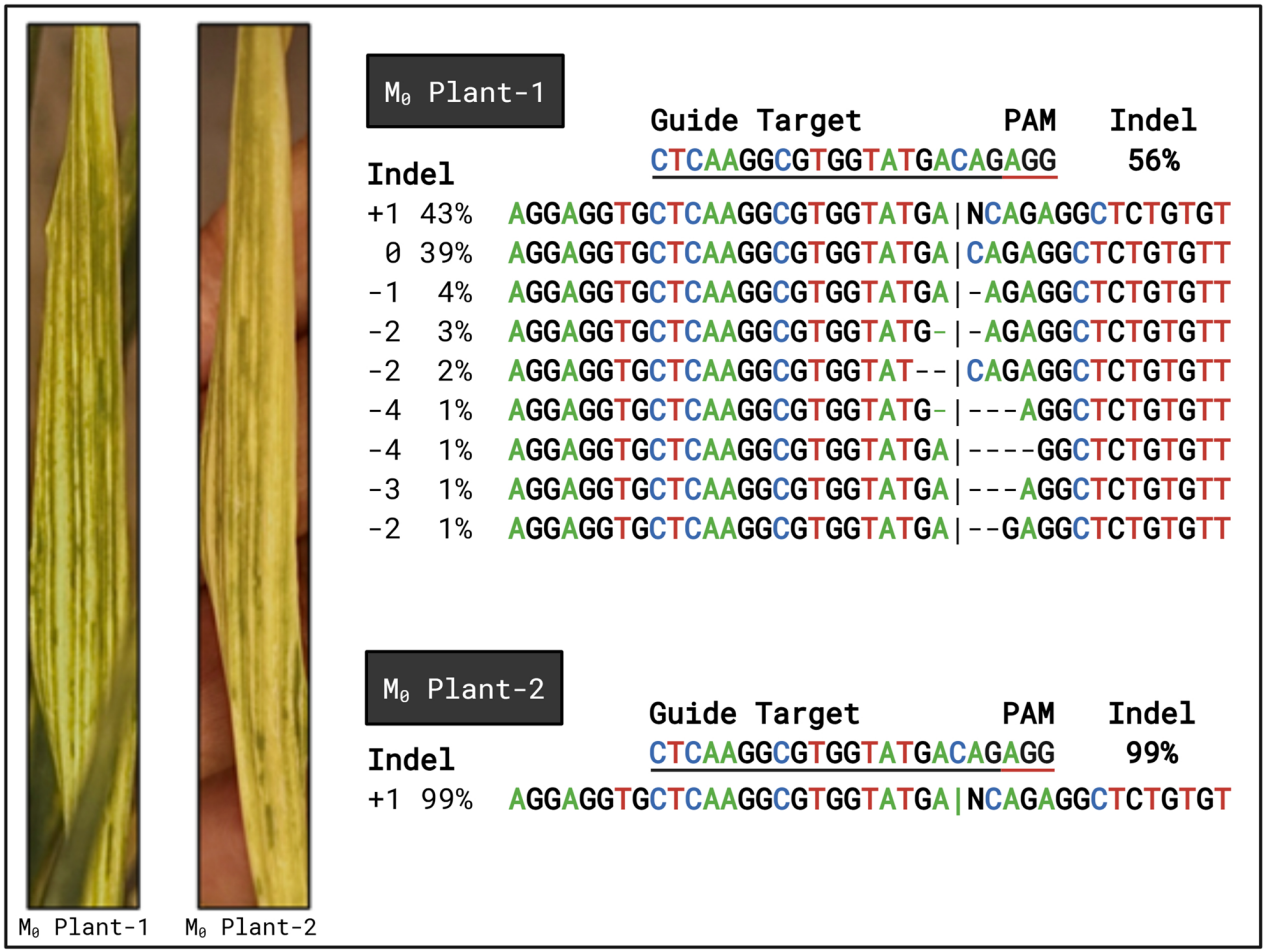
BSMV-delivered *CMF7* sgRNA induces albostrians-like chlorosis and striping in systemic barley leaves, with corresponding editing frequencies quantified from Sanger sequencing by ICE.​ Left: Representative systemic leaves from two rub-inoculated M₀ plants show variegated chlorosis consistent with loss of *CMF7* function in chloroplast development and the classic albostrians phenotype. Leaves derive from plants mechanically inoculated with BSMV vectors encoding the *CMF7* sgRNA.​ Right: ICE analysis of amplicon Sanger sequencing at the *CMF7* target with estimated indel frequencies

**Fig. 4:**
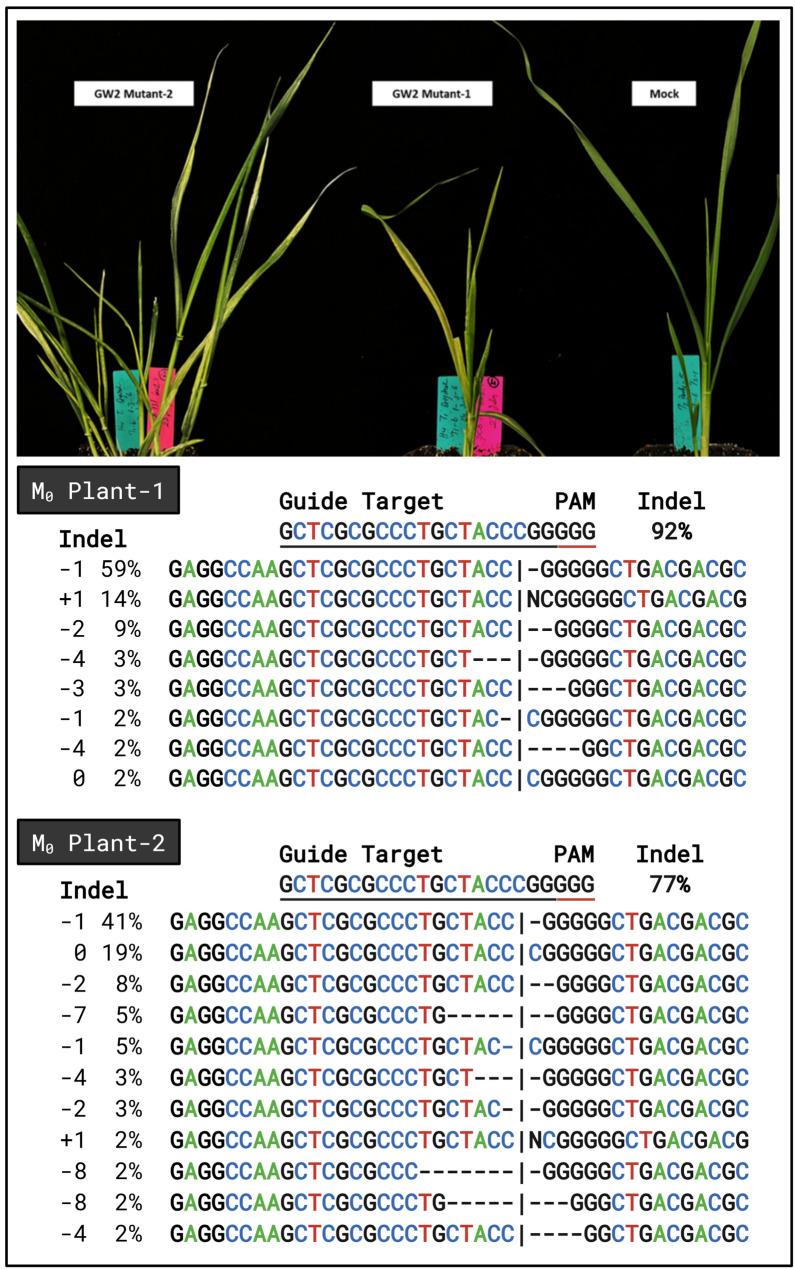
BSMV-delivered *GW2.1* sgRNA generates high-efficiency edits in barley with visible vegetative phenotypes and robust ICE-confirmed indel profiles.​ Top: Representative plants at the vegetative stage show two independent edited lines (*GW2.1* M_0_ plant-1 and *GW2.1* M_0_ plant-2) compared to a mock inoculated control, revealing altered vigor and leaf color relative to mock under identical growth conditions.​​ Bottom: ICE analysis for *GW2.1* target guide indicating on-target mutagenesis consistent with frameshift-enriched outcomes

**Fig. 5:**
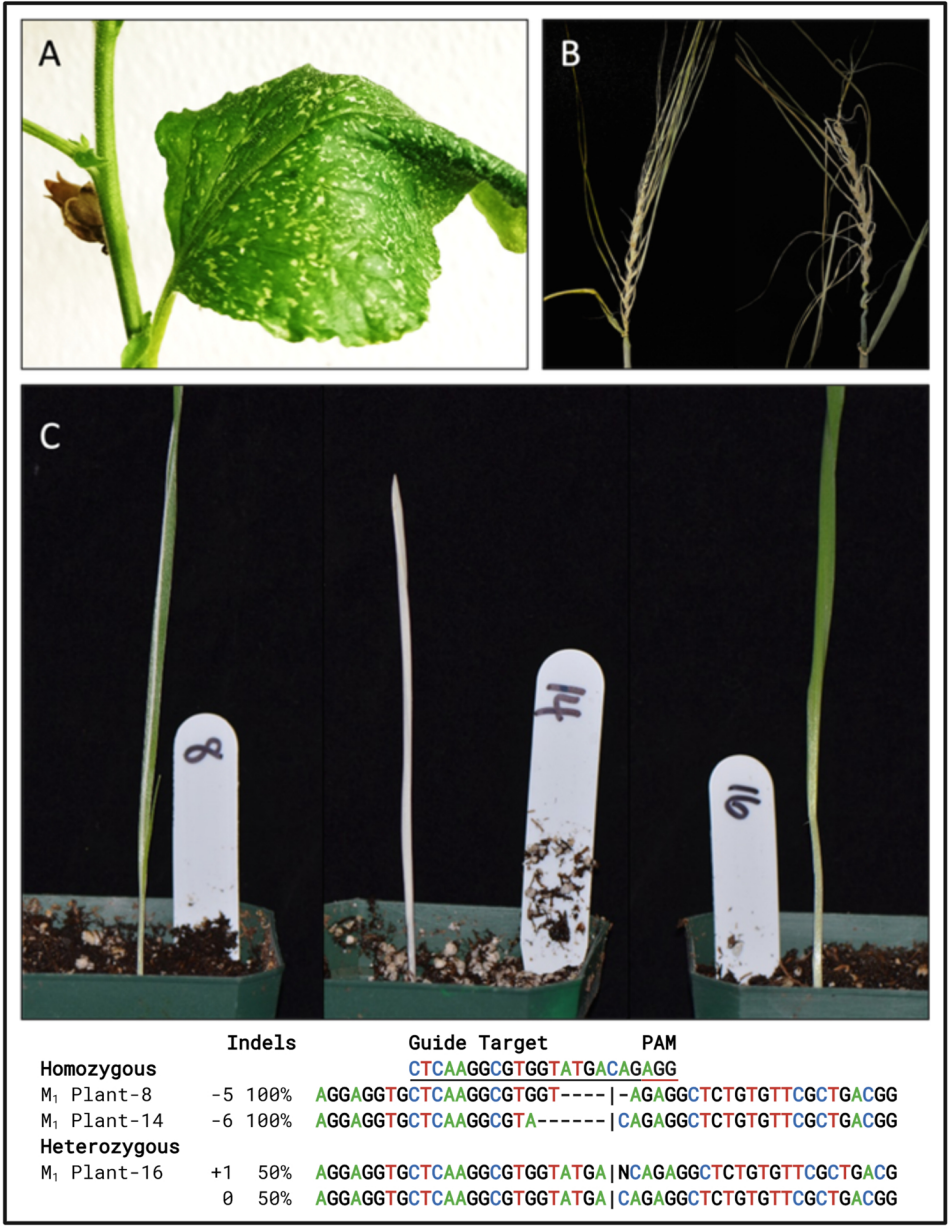
Heritability outcomes of BSMV-delivered sgRNAs targeting *PDS* in *N. benthamiana* and *CMF7* and *GW2.1* in barley. **A** Representative systemic leaf and seed pod of *N. benthamiana* at 60 days post infiltration showing robust *PDS* editing phenotype yet no evidence of heritable PDS bleaching in progeny despite strong somatic editing observed for VIGE systems in this host. PDS knockout yields albino or chimeric bleaching, enabling facile assessment of editing; here, bleaching was restricted to somatic tissues and did not transmit to the next generation (Fig. S6). **B** Representative barley spikes from M₀ plants edited at *GW2.1* using BSMV-delivered *GW2.1* sgRNA, illustrating failure to set filled seed and severe fertility defects in edited M₀ plants. Detailed sequencing analysis were shown in (Fig. S7). **C** Representative M_1_ seedlings harvested from two independent CMF7-edited M₀ barley lines show albino/variegated phenotypes consistent with heritable transmission. 24/30 seedlings exhibited mono- and biallelic, (non-chimeric) mutations. Sequencing analysis of *CMF7* mutations is shown here and in Fig. S4

To determine whether BSMV could induce comparable editing outcomes in a monocot system, we selected our best-performing expression vector and generated two γb-based sgRNA T-DNAs targeting barley *Chloroplast Maintenance Factor 7* (*CMF7*) and *Grain Width and Weight 2* (*GW2.1*), two well-characterized loci associated with leaf variegation and grain morphology, respectively. Viral sap was prepared by Agrobacterium-mediated infiltration of *N. benthamiana* plants, and sap extracts were subsequently used to rub-inoculate six Cas9-expressing transgenic barley lines for each target. Two out of six plants for each target demonstrated systemic viral infection by severe systemic viral symptoms from rubbed and upper, uninoculated leaves. Consistent with observations in *N. benthamiana*, BSMV spread efficiently throughout barley plants and induced high-frequency somatic mutations, with editing efficiencies reaching up to 100% at both target sites (Fig. 3 and Fig. 4). Mutation patterns included small insertions and deletions characteristic of CRISPR/Cas9 activity (Fig. 3 and Fig. 4). Editing of *HvCMF7* produced distinct green-white variegation phenotypes, whereas *HvGW2.1* editing resulted in visibly altered spike morphology and caused sterility (Fig. 3 and Fig. 5).

Barley plants that exhibit viral symptoms and higher editing frequencies for *HvCMF7* gene were grown to maturity. Approximately 15 seeds produced by each plant were germinated to assess the heritability of the BSMV induced mutations **(**Fig. 5 and Fig. S4**)** We anticipated observing white to pale yellow seedlings if any carried heritable loss-of-function mutations in the targeted genes. Remarkably, heritable mutations were detected in the M₁ progeny derived from only BSMV-sg*HvCMF7* infected barley plants (Fig. 5C, Fig. S4). Targeted sequencing of the progeny confirmed efficient transmission of edited alleles, 24 over 30 germinated seedlings carried mono- and bi-allelic mutations, with no chimeric mutation detected (Fig. S4**)**. RT-PCR assays verified the absence of viral RNA, indicating completely virus free progeny, and BSMV symptoms were not observed in any of the germinated barley seedlings (Fig. S5). In contrast, *N. benthamiana* progeny showed no evidence of heritable editing (Fig. S6), suggesting that BSMV replication or movement is restricted within the reproductive tissues of dicots despite high somatic editing efficiency.

## Discussion

Our study demonstrates that BSMV is a powerful platform for *in planta* delivery of CRISPR/Cas9 components in both dicot and monocot hosts. γb-based promoter designs, particularly direct fusion to the γb genomic RNA and duplicated γb subgenomic promoter, proved most efficient for sgRNA expression and editing activities. These optimized constructs induced high-frequency somatic mutations in transgenic *N. benthamiana* (up to 90%) and barley (up to 100%), approaching conventional tissue culture-based transformation efficiencies. The most striking finding is the host-specific boundary for BSMV-mediated germline transmission. Importantly, viral transmission and germline editing are distinct processes. Although BSMV was not detected in progeny of either barley or *N. benthamiana*, we recovered mono- and biallelic, non-chimeric mutations in barley offspring, indicating that genome editing occurred in germline precursor cells prior to seed formation. This provides indirect evidence that viral-delivered sgRNAs reached reproductive tissues without resulting in transmission of the virus to the next generation. In contrast, despite high somatic editing in *N. benthamiana*, the absence of heritable mutations suggests that BSMV-mediated delivery is restricted from germline or meristematic tissues in this host. These results highlight a host-dependent boundary between systemic infection and germline access. This critical distinction establishes actionable design rules for viral vector optimization balancing sgRNA abundance, viral fitness, and host compatibility. The host-specific heritability boundary observed here is consistent with dicot VIGE studies, where achieving germline access has typically required vectors with meristem tropism or engineered delivery strategies. In tomato, heritable genome editing has been obtained using TRV vectors carrying sgRNAs fused to mobile RNA sequences (FT or tRNA), yielding up to 86–100% edited progeny under optimized conditions [33]. In a separate approach, RNA virus–mediated delivery using PVX, together with co-delivery of developmental regulators or cytokinin treatments to enhance meristem infection, has also produced germline edits and homozygous knockout plants [12]. Additionally, sgRNA delivery to latent axillary meristematic cells combined with expression of a cytokinin biosynthesis gene to induce new meristem formation, has generated edited shoots and heritable mutants [11]. Collectively, these strategies demonstrate that overcoming germline barriers requires either vectors with appropriate tissue tropisms or hybrid approaches that promote infection or regeneration of meristematic lineage cells while mitigating localized antiviral defenses. Our findings complement recent BSMV-mediated genome editing studies in wheat and barley. In wheat, BSMV-delivered sgRNAs have yielded heritable editing frequencies ranging from 0.8 to 100%, depending on genotype, Cas9 expression level, sgRNA design, and the inclusion of tRNA or mobile RNA sequences [9]. The study reported that somatic editing frequency in M₀ wheat plants correlates positively with heritable mutation frequency in M₁ progeny, with the strongest correlation observed in upper systemic leaves [9]. This pattern is consistent with our observation that 60 dpi editing frequencies in *N. benthamiana* and barley best predicted vector performance. Multiplexed promoter and gene editing in wheat has also been achieved using pooled BSMV-sgRNAs, resulting in up to 61% of M₁ mutants with all six TaGW2 homeologs edited, and highlighting that high Cas9 expression levels are critical for robust BSMV-based editing [34].

In barley, BSMV-mediated somatic editing at *ASY1*, *MUS81*, and *ZYP1* loci with mean mutation efficiencies of 17–19% and successfully generated heritable edits at only *HvCMF7* locus (recreating the *albostrians* phenotype) were reported with somatic editing frequencies averaging 57% and individual plants reaching 94%. The study also noted that plants with intermediate to high somatic editing frequencies yielded heritable edits though the relationship between somatic and germline editing was not strictly linear [10]. Our results with *HvCMF7* in barley align closely with these findings, achieving 100% editing frequencies in M_0_ plant and recovering virus-free edited progeny only for *HvCMF7* target directly from seed.​​ Unexpectedly, M_0_ plants infected with BSMV carrying *HvGW2.1*-targeting sgRNA exhibited complete sterility, a phenotype likely resulting from the synergistic interaction of both BSMV viral infection and induced *HvGW2.1* loss-of-function mutations. While published stable CRISPR knockouts of *HvGW2.1* report “low grain setting” with reduced fertility characterized by fewer grains per spike, our BSMV-mediated approach produced more severe reproductive failure with completely empty spikes and zero seed production [30]. This enhanced severity can be attributed to the combination of two distinct but potentially overlapping mechanisms: (1) robust BSMV viral infection, which replicates in developing reproductive tissues and induces systemic stress responses that impact meristem function and assimilate allocation, and (2) strong somatic mutations of *HvGW2.1* induced by high-frequency which disrupts the gene’s critical role in maintaining spikelet meristem identity and floret viability during the grain-setting phase.

In sorghum, FoMV-delivered sgRNAs induced somatic genome editing, with mutation frequencies reaching up to 60% at *PDS*, *MgCh*, and *Lw1* orthologs [2]. BSMV failed to infect sorghum under the conditions tested, highlighting host range constraints that limit its applicability to certain cereals (barley, wheat, oat). FoMV’s broader monocot host range and compatibility with sorghum, maize, and potentially switchgrass position it as a complementary platform to BSMV for virus-induced genome editing in monocots.​ However, FoMV’s limited access to meristematic tissues reinforces BSMV as a promising transformation-free tool for monocot cereals, while defining a clear limitation for dicot applications. This reveals fundamental differences in viral access to reproductive lineages within monocots and dicot hosts with important implications for platform selection​.

The subgenomic promoter elements strength ranking, direct γb fusion > duplicated γb SGP > β1 SGP > β2 SGP, reflects native BSMV RNA abundance patterns [26–28]. The constitutively high γb expression, driven by its critical roles in viral replication and movement, made direct γb coupling optimal for maximizing sgRNA delivery without compromising movement protein stoichiometry. The duplicated γb SGP design further amplified sgRNA output while maintaining systemic spread. In contrast, β1-driven constructs exhibited delayed kinetics consistent with the transient, temporally regulated nature of β1 expression, suggesting utility for applications requiring moderate or time-resolved editing. The failed β2-length construct and reduced performance of truncated β2 designs indicate that this promoter context is suboptimal for sgRNA expression in our system, despite functional activity at 60 dpi for the truncated variant. These results establish clear design hierarchies for BSMV vector optimization based on native promoter biology. The promoter and transcript-architecture optimizations presented here also define the expression level necessary for next-generation BSMV systems that co-deliver editing nucleases from the viral genome. Recent advances in hypercompact CRISPR effectors, including engineered Cas12f variants and miniature TnpB enzymes, have demonstrated that small editors (~ 400–600 amino acids) can function in plant cells at useful levels, and proof-of-concept viral co-delivery of compact nucleases has achieved transgene-free germline editing in Arabidopsis and tomato [20, 21]. Translating these approaches to BSMV in cereals is now feasible given the promoter performance benchmarks established here: the γb-fusion and duplicated γb SGP architectures support high sgRNA output while preserving robust systemic movement, suggesting that similar designs could accommodate compact nucleases when optimized for codon usage, RNA structure, and expression timing.​​ Such systems would enable fully transformation-independent genome editing in cereals, delivering both nuclease and guide from a transient viral vector to generate virus-free edited progeny without any stable transgene integration or tissue culture. This capability would dramatically accelerate cereal functional genomics and trait development, particularly in recalcitrant genotypes where transformation remains a bottleneck.

Our findings here establish actionable design rules: (1) prioritize direct γb fusion or duplicated γb SGP for maximum somatic and heritable editing; (2) use β1 SGP for intermediate or time-resolved expressions and editing; (3) restrict BSMV to somatic editing in dicots unless combined with regeneration or alternative germline-targeting strategies. Future work should focus on: (i) testing whether mobile RNA elements (FT, tRNA) to induce BSMV-mediated heritable editing in dicots (ii) expanding host range through chimeric vectors or movement protein engineering; (iii) evaluating BSMV performance in other cereals (rye or triticale); and (iv) engineering BSMV to co-deliver compact nucleases alongside sgRNAs to enable heritable editing in recalcitrant crop species​.

## Conclusions

BSMV’s compatibility with mechanical inoculation and broad host range within cereals make it an attractive platform for rapid prototyping of edits across multiple species, reducing the cycle time from target identification to phenotypic validation from months to weeks.​​ Our study establishes BSMV as a versatile, tissue culture-free platform for somatic genome editing in both barley and *N. benthamiana*, and as a practical tool for heritable editing in barley where viral access to reproductive lineages is permissive. The host-specific boundary for germline transmission revealed here clarifies where BSMV is optimally suited and where alternative vectors or hybrid strategies will be required, guiding rational platform selection for diverse crop species and applications. The promoter optimizations presented here provide a foundation for engineering BSMV systems that co-deliver compact nucleases, moving toward a future where tissue culture-free, transformation-independent and transgene-free genome editing becomes routine in cereal crop improvement.​​

## Supplementary Information

Supplementary Material 1.

## Data Availability

All relevant data supporting the findings of this study are available within the manuscript and its Supporting Information File. The plant materials used in this study were located at the University of Saskatchewan, Saskatoon, Saskatchewan, Canada, and at the National Research Council Canada, Saskatoon, Saskatchewan, S7N 0W9, Canada. Plasmids used in this study have been deposited in the Addgene repository.
