# Supplementary material for: BSMV-mediated genome editing exhibits host-specific heritability: germline transmission in barley and somatic edits in Nicotiana benthamiana
Source: BMC Plant Biol. 2026 May 12;26:1132. doi: 10.1186/s12870-026-08866-3 (PMC13335176; doi:10.1186/s12870-026-08866-3)
Supplement: Supplementary file 1 — Supplementary Material 1. [file 12870_2026_8866_MOESM1_ESM.pdf]

BSMV-Mediated Genome Editing Exhibits Host-Specific Heritability: Germline Transmission in Barley and Somatic Edits in *Nicotiana benthamiana*

Pankaj K. Bhowmik[1], John T. Williams[2,3] Brittany Polley[1], Naichong Chen[2,3], Naga Rajitha Kavuri[2,3], Wen Zang[4], Abdellah Barakate[5], Hui Yang[1], Murali Krishna Narra[1], Aaron D. Beattie[4], Colby Starker[6], Daniel F. Voytas[6] and Can Baysal[2,3]*

[1]National Research Council of Canada, Saskatoon, SK S7N 0W9, Canada.
[2]Department of Horticultural Sciences, University of Florida, Gainesville, FL, 32611, USA.
[3]Crop Transformation Center, University of Florida, Gainesville, FL, 32611, USA.
[4]Department of Plant Sciences, University of Saskatchewan, Saskatoon, SK S7N 5A8, Canada.
[5]Cell and Molecular Sciences, The James Hutton Institute, Invergowrie, Dundee, Scotland, UK.
[6]Department of Genetics, Cell Biology and Development, University of Minnesota, St. Paul, MN 55108, USA.
*Correspondence email: can.baysal@ufl.edu

Supplementary Information

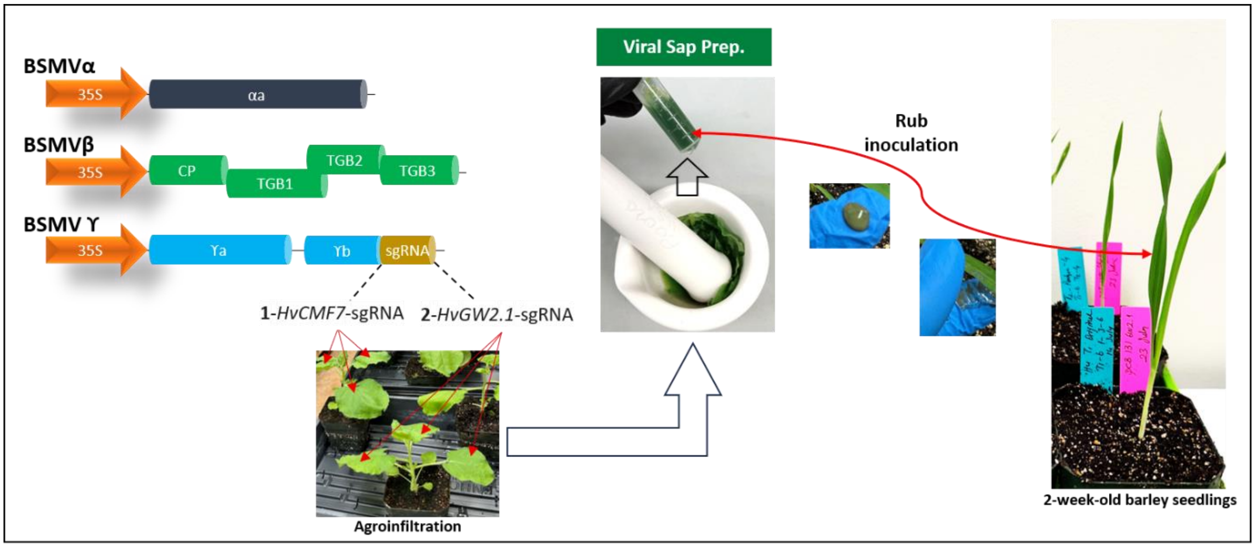

Fig. S1. Preparation of viral sap for mechanical (rub) inoculation. *Nicotiana benthamiana* leaves were agroinfiltrated with BSMV α, β, and γ constructs (individual plants for each γ design), followed by tissue harvest at 10 dpi. Infected tissue was homogenized in phosphate buffer to prepare viral sap, which was then mixed with an abrasive (celite or silicon carbide). The sap was used for mechanical inoculation of barley seedlings at the two–three leaf stage.

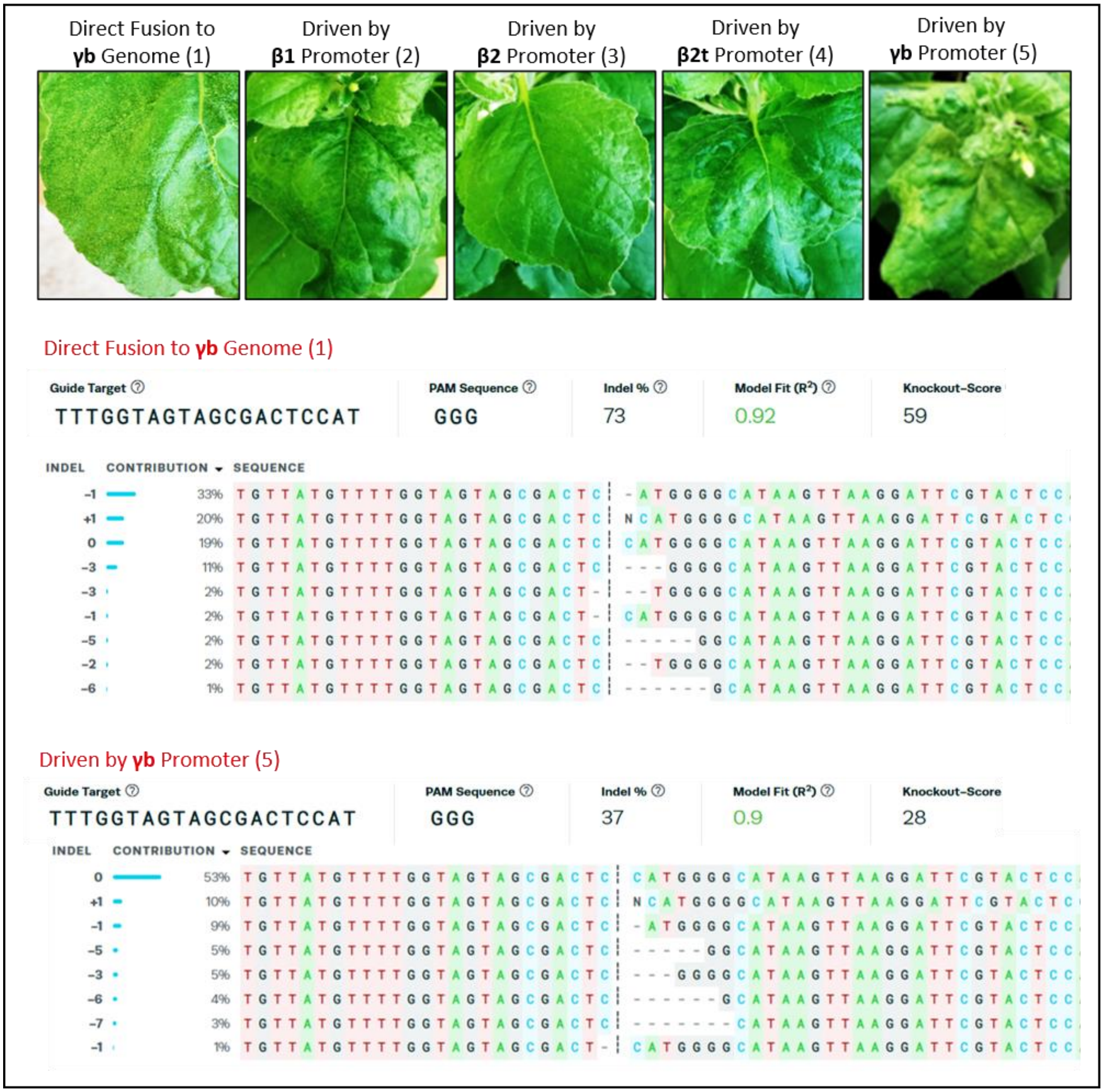

Fig. S2. Detailed analysis of BSMV induced mutations on systemic leaves of *N. benthamiana* at 30 dpi. Sanger sequencing and ICE indel analysis of the *NbPDS* target site from systemic leaves at 30 days post-inoculation (dpi). Mutation profiles illustrate editing events across different subgenomic promoter architectures, highlighting higher editing efficiency in two γb-based constructs.

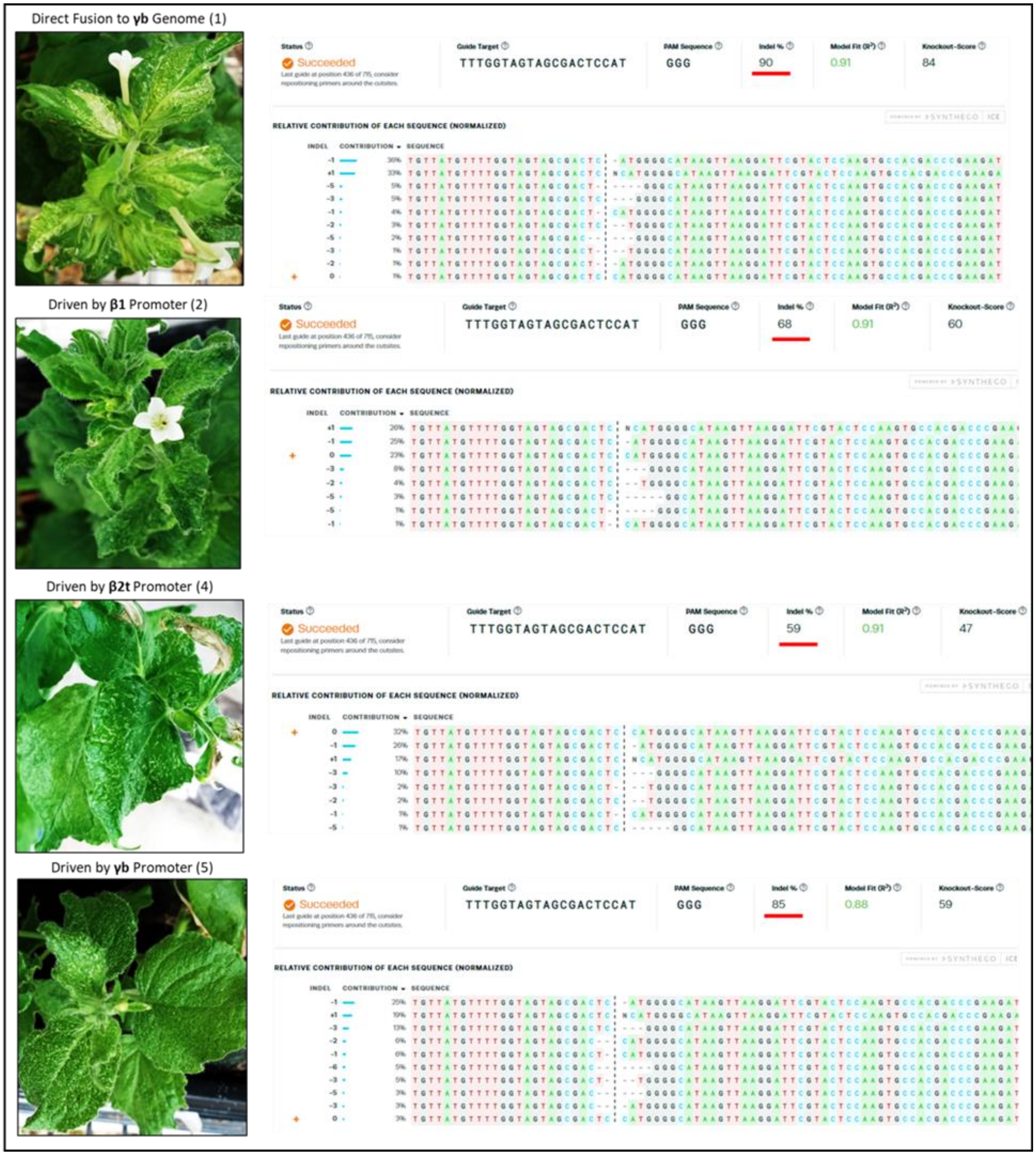

Fig. S3. Detailed analysis of BSMV induced mutations on systemic leaves of *N. benthamiana* at 60 dpi. Sanger sequencing and ICE indel analysis of *NbPDS* target regions at 60 dpi. Increased mutation frequency and diversity relative to 30 dpi confirm progressive sgRNA accumulation and time-dependent enhancement of editing efficiency across functional constructs.

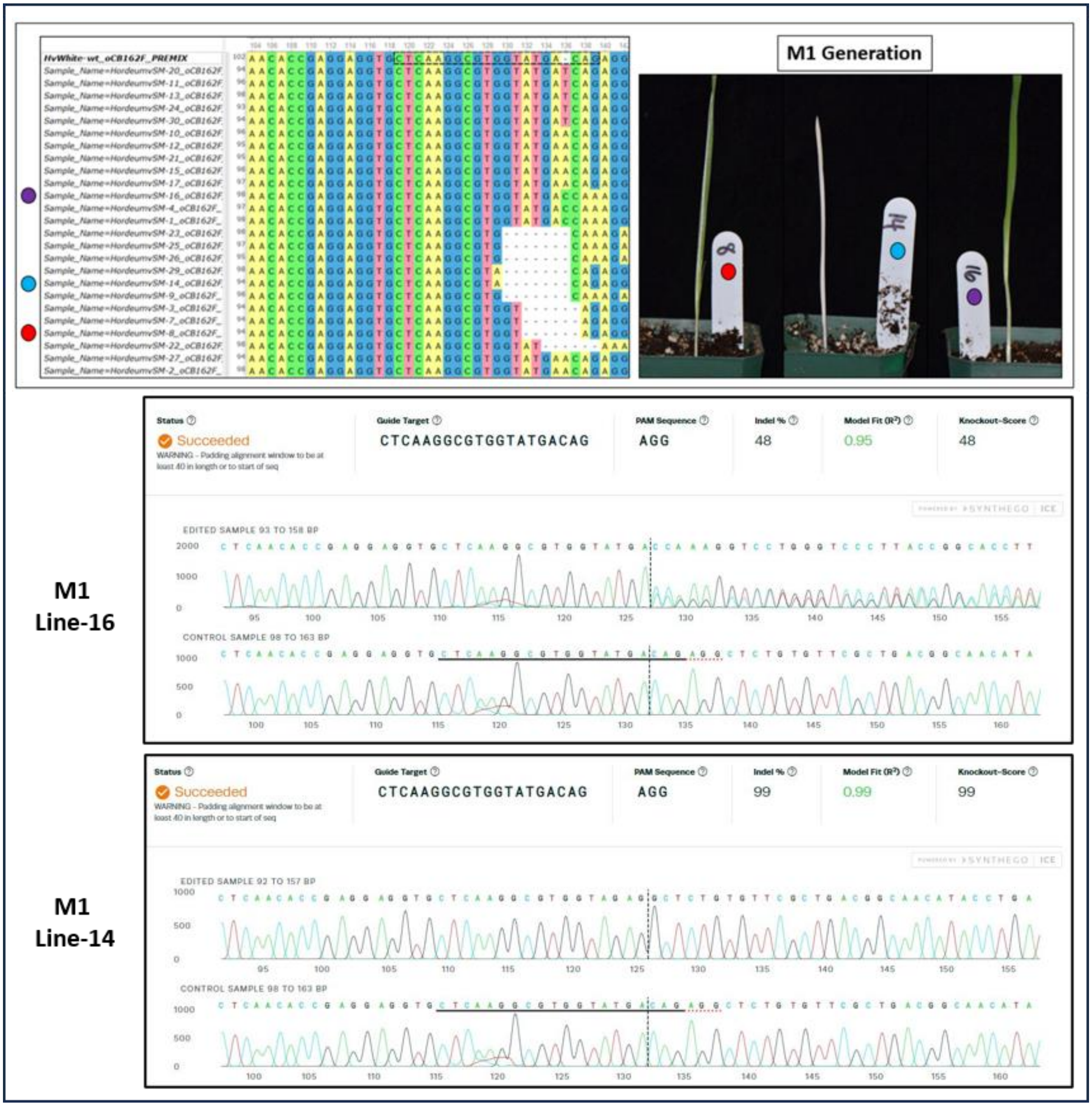

Fig. S4. Detailed analysis of BSMV-induced heritable mutagenesis in *HvCMF7*. Genotyping and sequencing of *HvCMF7* alleles in M_1 progeny derived from infected barley plants. Representative chromatograms show mono- and biallelic, non-chimeric mutations, confirming efficient germline transmission of edits. Corresponding phenotypes (albino/variegated) are consistent with loss-of-function mutations.

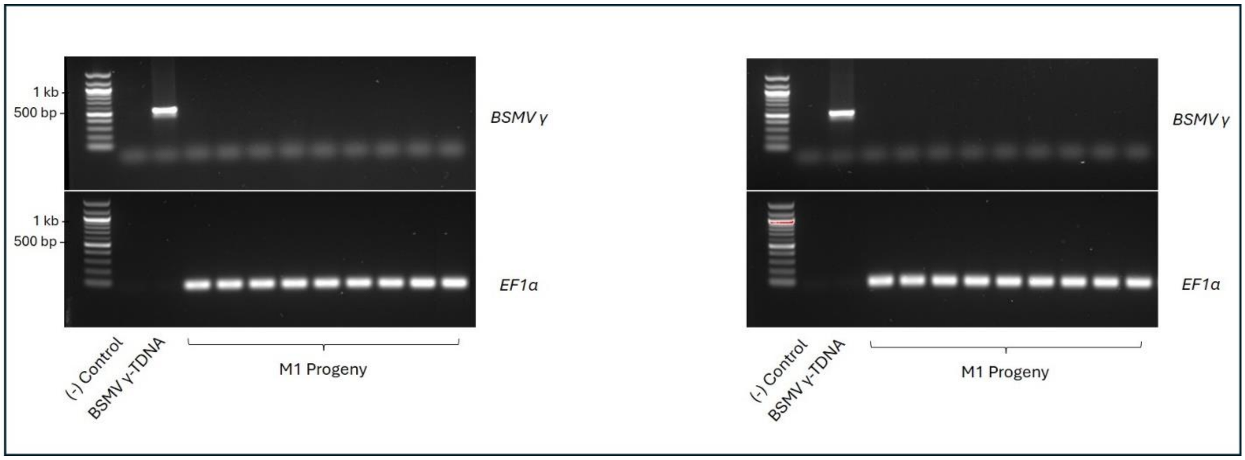

Fig. S5. Analysis of seed transmission of BSMV in M_1 progeny by RT-PCR. RT-PCR detection of BSMV RNA in M_1 seedlings derived from infected barley plants. No viral RNA was detected in 18 randomly selected samples, indicating absence of seed transmission and confirming recovery of virus-free edited progeny.

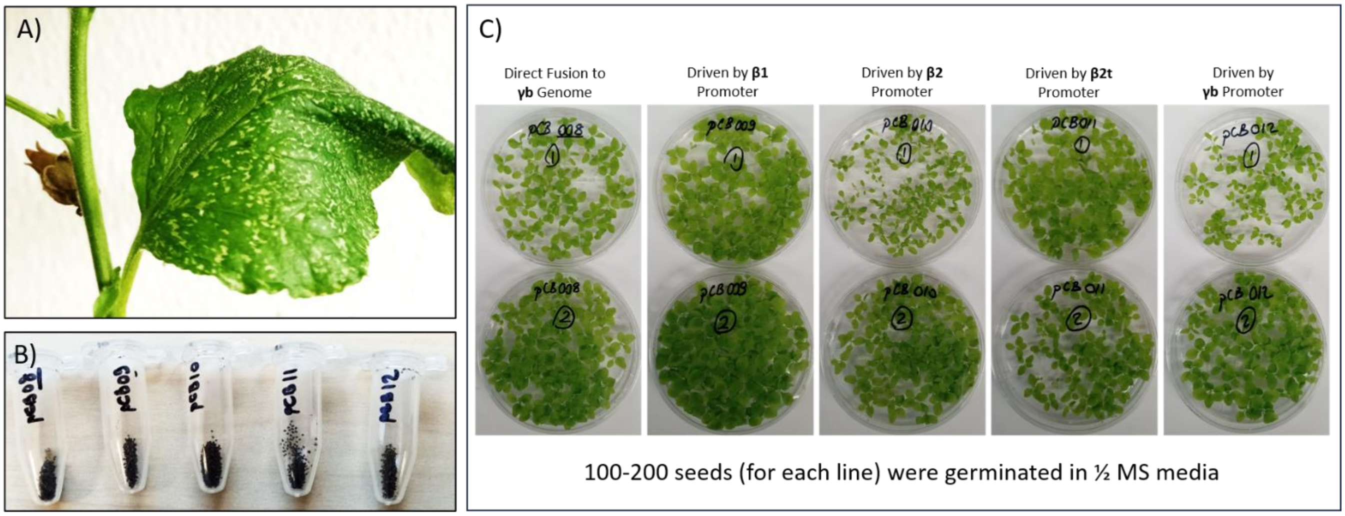

Fig. S6. Heritability analysis of BSMV induced mutations in *N. benthamiana*. Assessment of M_1 progeny derived from infected Cas9-expressing *N. benthamiana* plants. Despite high somatic editing in M_0 plants, no heritable PDS mutant phenotypes (photobleaching) were observed, indicating failure of germline transmission in this host.

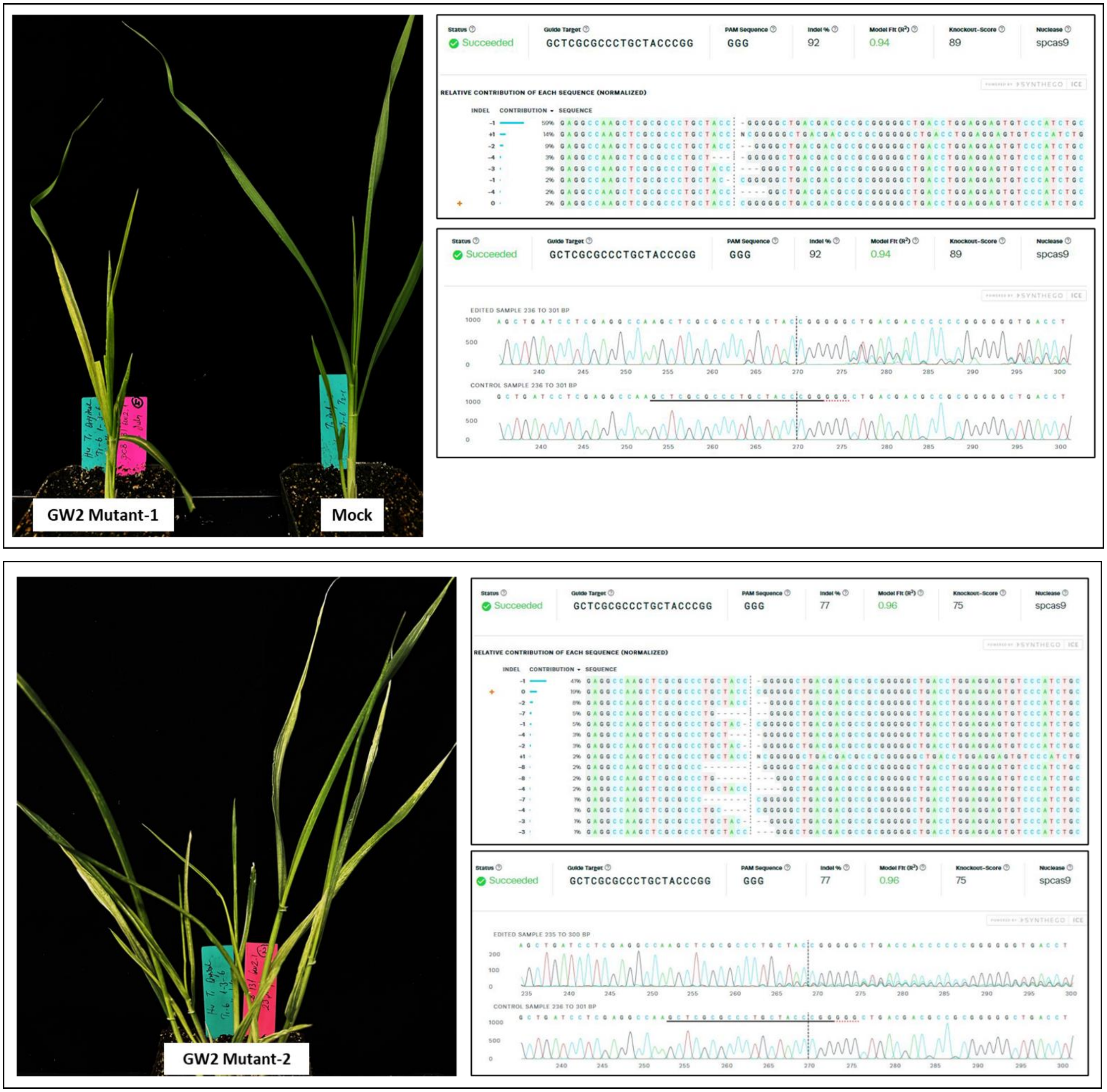

Fig. S7. Detailed analysis of BSMV induced somatic mutagenesis in *HvGW2* at 10 dpi. Sanger sequencing, ICE indel analysis, and representative chromatograms of the *HvGW2.1* target site in two infected M_0 plants.

Table S1.

OLIGOS USED IN THIS STUDY	
**Annealed Oligos with SapI overhangs used for *NbPDS* sgRNA Cloning in (pYL157) BSMV Ɣ Genome along with Subgenomic Promote[illegible] (RC: Reverse complement)**	
**1-*NbPDS* Forward**	CTATTTGGTAGTAGCGACTCCAT
**2-*NbPDS* RC**	AACATGGAGTCGCTACTACCAAA
**3-BSMV pβ1+*NbPDS* Forward**	CTAAGTTTTTGCTTTTTACGCGTTAACTAGATGTTTGGTAGTAGCGACTCCAT
**4-BSMV pβ1+*NbPDS* RC**	AACATGGAGTCGCTACTACCAAACATCTAGTTAACGCGTAAAAAGCAAAAACT
**5-BSMV pβ2+*NbPDS* Forward**	CTATCAGGGCCGACGCGGAAATTCGTCAAGCATTCCTGACAGGTGATATTGACTTGAGCTCTAAGGCGTTT[illegible] TAGTAGCGACTCCAT
**6-BSMV pβ2+*NbPDS* RC**	AACATGGAGTCGCTACTACCAAACGCCTTAGAGCTCAAGTCAATATCACCTGTCAGGAATGCTTGACGAAT[illegible] CGCGTCGGCCCTGA
**7-BSMV pβ2t+*NbPDS* Forward**	CTATCAGGGCTGACAGGTGATATTGACTTGAGCTCTAAGGCGTTTGGTAGTAGCGACTCCAT
**8-BSMV pβ2t+*NbPDS* RC**	AACATGGAGTCGCTACTACCAAACGCCTTAGAGCTCAAGTCAATATCACCTGTCAGCCCTGA
**9-BSMV pYb+*NbPDS* Forward**	CTATGGTGAACTCTAGGTCCTGATGTTTTGGTAGTAGCGACTCCAT
**10-BSMV pYb+*NbPDS* RC**	AACATGGAGTCGCTACTACCAAAACATCAGGACCTAGAGTTCACCA
**Annealed Oligos with AarI overhangs used for *HvCMF7* and *HvGW2.1* sgRNA Cloning in (pGY036) BSMV Ɣ Genome**	
**11-*HvCMF7*-Forward**	ATTGCTCAAGGCGTGGTATGACAG
**12- *HvCMF7*-RC**	AAACCTGTCATACCACGCCTTGAG
**13-*HvGW2.1*-Forward**	ATTGGCTCGCGCCCTGCTACCCGG
**14- *HvGW2.1*-RC**	AAACCCGGGTAGCAGGGCGCGAGC
**Oligos used to PCR amplify target regions**	
**15-*NbPDS*-F (743bp)**	GCTTTGCTTGAGAAAAGCTCTC
**16-*NbPDS*-R**	CCAATGGTTTAGTTGGGCGTG
**17- *HvCMF7*-F (300bp)**	GCGTTGAAGGACATAGCTGC
**18- *HvCMF7*-R**	AGTTCTAATGCAGTGCAGGC
**19- *HvGW2.1*-F (550bp)**	CAGGGTAATCCCACCTCGCCTCGG
**20- *HvGW2.1*-R**	CTCACGGCAGCGGCACTCTACG
**Sequence elements controlling expression of *Barley stripe mosaic virus* subgenomic RNAs**	
**β1 Promoter (pβ1)**	AGTTTTTGCTTTTTACGCGTTAACTAGATG
**β2 Promoter (pβ2)**	TCAGGGCCGACGCGGAAATTCGTCAAGCATTCC**TGACAGGTGATATTGACTTGAGCTCTAAGGCG**
**β2 Truncated Promoter (pβ2t)**	TCAGGGC**TGACAGGTGATATTGACTTGAGCTCTAAGGCG**
**Yb Promoter (pγb)**	TGGTGAACTCTAGGTCCTGATGT
